# A Comprehensive Analysis of the 9-Cis Epoxy Carotenoid Dioxygenase Gene Family and Their Responses to Salt Stress in *Hordeum vulgare* L.

**DOI:** 10.3390/plants13233327

**Published:** 2024-11-27

**Authors:** Fatima Omari Alzahrani

**Affiliations:** Department of Biology, Faculty of Sciences, Al-Baha University, Al-Baha 65729, Saudi Arabia; drfatimaomari@gmail.com or fsalomari@bu.edu.sa

**Keywords:** NCED, abscisic acid (ABA), salt stress, phylogeny, salinity

## Abstract

Barley (*Hordeum vulgare* L.) is among the earliest crops to be cultivated and is also considered a crucial staple crop. Nevertheless, the negative effects of abiotic stress on both the quality and productivity of barley are significant. Nine-cis-epoxycarotenoid dioxygenases (NCEDs) are rate-limiting enzymes in plants that cleave carotenoids and produce abscisic acid (ABA). The poor utilization of barley *NCED*s in stress-resistant genetic breeding is due to the lack of appropriate information about their potential function in abiotic stress. The current study revealed five *NCED* genes in the barley genome (*HvNCED1*—*HvNCED5*), which are distributed unevenly on barley chromosomes. The PF03055 domain is present in all *HvNCED*s, and they encode 413~643 amino acids. Phylogenetic analysis showed that *NCED* genes were categorized into three distinct clades, confirming the homology of *NCED* genes between *H. vulgare* L., *Arabidopsis thaliana* L., and *Oryza sativa* L. Expression analysis revealed that *HvNCED*1 is significantly upregulated under high salt stress, indicating its potential role in enhancing salt tolerance. In contrast, *HvNCED*3 and *HvNCED*4 exhibited downregulation, suggesting a complex regulatory mechanism in response to varying salt stress levels. These findings will enhance our comprehension of the genetic composition and evolutionary development of the *HvNCED* gene family and provide a basis for future research on their role in response to salt-induced stress.

## 1. Introduction

Abscisic acid (ABA) is a crucial phytohormone that has a substantial impact on the development and growth of plants, particularly in response to biotic or abiotic stress conditions [1,2,3,4,5,6,7,8]. Plant growth and yield are constrained by environmental conditions such as low temperature, drought, and soil salt. In higher plants, ABA is crucial for improving tolerance to a variety of environmental stressors [9,10,11,12,13]. In higher plants, ABA is synthesized by an indirect process involving the breakdown of carotenoids [14,15]. One of the main problems limiting crop yield is salinity, which leads to land abandonment for agriculture in arid and semi-arid regions of the world [16]. Controlling the metabolism and signaling of several plant hormones has been a primary biotechnological goal in the quest to create plants that are more resilient to stress [17,18,19].

Carotenoids, which are derived from isoprene, have been a subject of scientific interest since their discovery in the 19th century [20]. Their mysterious variety is revealed through the examination and categorization of over 700 unique types throughout time, uncovering a complex array of possible advantages yet to be discovered [21]. These chemicals serve a variety of biological roles in a wide range of organisms, including bacteria and plants. Carotenoids are crucial secondary pigments that enable photosynthetic organisms to capture light more effectively and prevent photooxidation [22]. Furthermore, the cleavage of carotenoids results in the production of specific apocarotenoids, which have a role in the regulation of plant growth and development, as well as the plant’s reaction to both biotic and abiotic causes of stress [23]. A number of enzymes known as carotenoid cleavage oxygenases (CCOs) are responsible for the degradation of carotenoids, which are naturally occurring pigments that contribute color to a variety of fruits, vegetables, and florals [24]. CCOs consist of two types of enzymes: carotenoid cleavage dioxygenases (CCDs) and 9-cis-epoxycarotenoid dioxygenases (NCEDs). These enzymes play a crucial role in the conversion of carotenoids into apocarotenoids, which undergo degradation [24,25]. In order to create apocarotenoids, CCOs require oxygen to cleave the carotenoid molecule [26].

The NCED enzymes catalyze the oxidative cleavage of 9-cis-epoxycarotenoids, resulting in the production of xanthoxin. This process is both the initial and the rate-limiting step in the biosynthesis of ABA. Xanthoxin is transported from the plastids to the cytosol and undergoes conversion to ABA by the action of abscisic aldehyde [27].

The plasticity of plants and their capacity to respond to stressful conditions are governed by a network of cross-signaling cascades involving many classes of phytohormones, including ABA, which play a crucial role in the coordination of growth and development [28]. ABA is recognized for its role in plant responses to environmental stressors, including drought and salinity. Mutants with deficient ABA production or perception exhibit heightened sensitivity to environmental fluctuations, whereas transgenic plants that synthesize elevated quantities of this hormone demonstrate greater tolerance to such abiotic stress compared to the wild type [29]. ABA can initiate its de novo synthesis in root tissues in response to stress such as drought and salinity. This molecule is mostly carried to the leaves, where it induces stomatal closure, hence regulating the transpiration rate and maintaining cell turgor [30].

In 1997, Schwartz et al. identified the first CCO gene, the vp14 gene, which was isolated from maize [31]. Tan et al. (2003) identified nine homologs of vp14 in *Arabidopsis thaliana* [32]. Four genes encoded CCDs (CCD1, CCD4, CCD7, and CCD8), while five encoded NCEDs (NCED2, NCED3, NCED5, NCED6, and NCED9) [33]. The CCO orthologs found in other plants were subsequently given names based on those found in Arabidopsis [34,35]. The *CCO* gene family has been extensively studied in multiple species [34,36,37,38,39,40,41,42]. Subsequently, the *NCED* gene family was identified and investigated in numerous plant species, including Arabidopsis, cotton, avocado, cowpea, kiwifruit, and grape [32,43,44,45,46,47].

Barley is a highly cultivated cereal crop and is highly valued for its food and beverage, livestock feed, and brewing properties [48,49]. It is a highly stress-tolerant crop, exhibiting resilience to many environmental stresses such as salt and low temperature. This makes it a valuable resource for genetic enhancement against both biotic and abiotic stressors.

Conducting research on *NCED* genes could provide a deeper understanding of their precise functions in barley’s physiological activities. In addition, it would enhance our comprehension of barley development at a genetic level and offer opportunities to enhance barley through breeding and genetic engineering methods. The main goal of the current study is to comprehensively identify the *NCED* genes in the barley genome. In addition, we utilized a range of bioinformatics methods to investigate their functions. The study conducted in this research will provide a comprehensive analysis and description of the genes, which will be used as a basis for the cloning and functional examination of these genes.

## 2. Results

### 2.1. Identification and Sequence Analysis of the NCEDs Family Genes in H. vulgare

An analysis was conducted on the *H. vulgare* genome using the HMM profile “PF03055” in order to identify the *HvNCED* genes. The genome of *H. vulgare* revealed five potential HvNCED proteins and their corresponding encoding genes (Table 1). The encoded proteins exhibited a range in length, spanning from 413 to 643 amino acids. The isoelectric points (Ip) of the HvNCEDs varied between 5.34 and 7.32. The molecular weight varied between 44,581 kDa and 69,174 kDa. Through subcellular localization predictions, it was concluded that HvNCED1 is located in the plastid, HvNCED2 and HvNCED5 are located in the membrane, and HvNCED3 and HvNCED4 are located in the cytoplasm.

### 2.2. Phylogenetic Analysis, Gene Duplication, Chromosomal Location, and Cis-Regulatory Element Analysis in the Promoter Regions of HvNCED Genes

**Figure 1:**
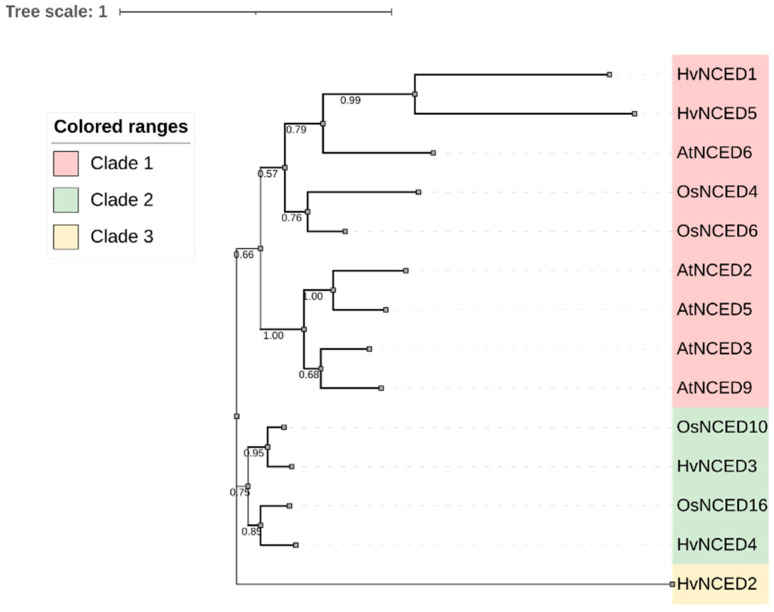
The phylogenetic tree of the *HvNCED* gene family, along with sequences from *Arabidopsis thaliana*, *Oryza sativa*, and *Hordeum vulgare.* The clades are represented using a range of distinct colors. The values of the bootstraps are provided.

The phylogenetic study of NCEDs in *A. thaliana*, *O. sativa*, and *H. vulgare* revealed that the NCEDs were classified into three primary clades, as shown in Figure 1. Clade 1 consists of two subclades. The first subclade includes HvNCED1 and HvNCED5, which have a phylogenetic relationship with AtNCED6 from *A. thaliana* and OsNCED4 and OsNCED6 from *O. sativa.* The second subclade solely contains NCEDs from *A. thaliana*, specifically AtNCED2, AtNCED5, AtNCED3, and AtNCED9. The second clade consists of NCEDs from *O. Sativa* and *H. vulgare*, specifically OsNCED10 with HvNCED3 and OsNCED16 with HvNCED4. The third clade exclusively consists of HvNCED2. 

**Figure 2:**
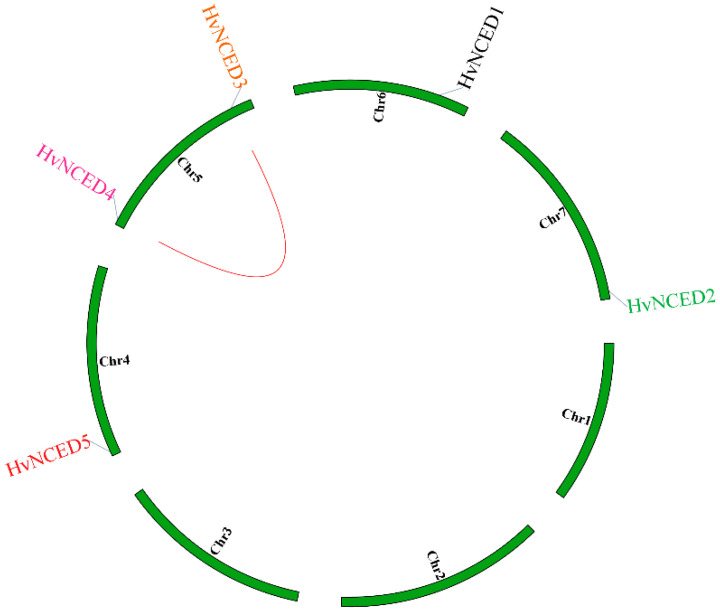
Chromosomal locations and gene duplication of the *HvNCED* gene family. The red line indicates the synteny between HvNCED3 and *HvNCED4* genes.

**Figure 3:**
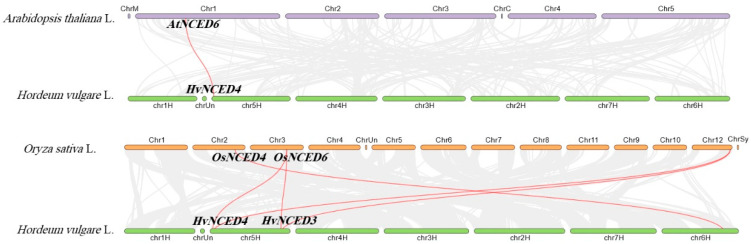
The synteny analysis of the *NCED* gene family members in three plant species: *Arabidopsis thaliana*, *Oryza sativa*, and *Hordeum vulgare*. The background of the image displays grey lines that show the synteny of the complete genome, whereas the red lines specifically demonstrate the synteny of the *NCED* genes.

The five *HvNCED* genes are distributed throughout four chromosomes. Chromosome 5 has two *NCED* genes, namely *HvNCED3* and *HvNCED4*. Chromosome 4 harbors the *HvNCED5* genes, whereas Chromosomes 6 and 7 possess the *HvNCED1* and *HvNCED2* genes, respectively. The *HvNCED* genes were annotated on the physical map of the *H. vulgare* chromosomes, utilizing position information acquired from NCBI. The map was then visualized using TBtool (Figure 2). Using synteny analysis, it seems probable that the genes *HvNCED3* and *HvNCED4* demonstrate segmental duplication, as shown in Figure 2. To examine evolutionary constraints and selection pressures on the *HvNCED* genes, we computed Ka, Ks, and the Ka/Ks ratio for the predicted homologous *HvNCED* gene pairs (as determined by the phylogenetic tree). The values of Ka, Ks, and Ka/Ks are provided in Table 2. A comparative analysis was conducted to investigate the association between *NCED* genes in three plant species: *A. thaliana*, *O. sativa*, and *H. vulgare* and revealed the presence of gene pairs consisting of *AtNCED6* and *HvNCED4*. Furthermore, there is an apparent synteny observed between *HvNCED1* and *OsNCED4*, and synteny between *HvNCED4* and *OsNCED10, OsNCED16*, and synteny between *HvNCED3* and *OsNCED10, OsNCED16* (Figure 3).

**Figure 4:**
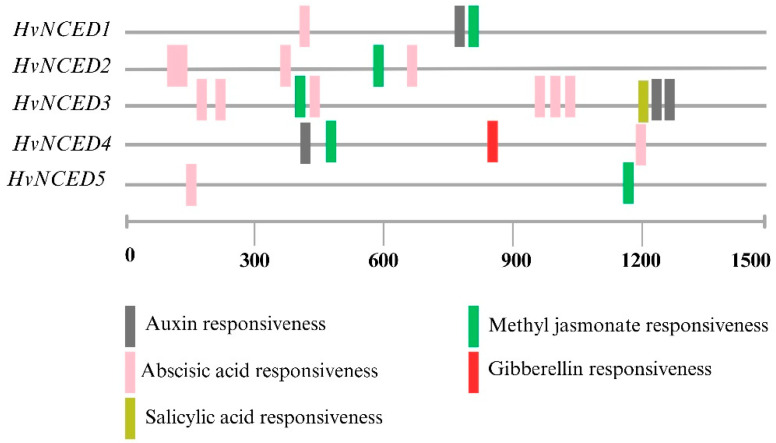
The cis-element analysis of *HvNCEDs*.

The findings reveal that several *HvNCED* genes are linked to a range of cis-acting elements and regulatory responses that are related to hormones. *HvNCED1*, *HvNCED2*, *HvNCED3*, *HvNCED4*, and *HvNCED5* were found to contain various hormone-related responsive elements such as ABA, auxin, gibberellin, salicylic acid, and methyl jasmonate (MeJA), as shown in Figure 4.

### 2.3. Gene and Protein Characteristics

**Figure 5:**
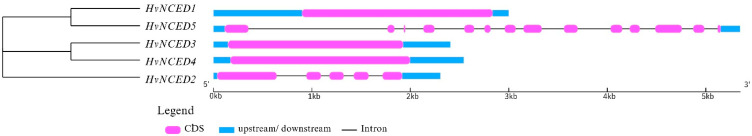
The *HvNCED* genes’ intron/exon structures are shown. Exons are represented by the pink boxes, introns by the black lines, and untranslated regions (UTRs) by the blue boxes.

**Figure 6:**
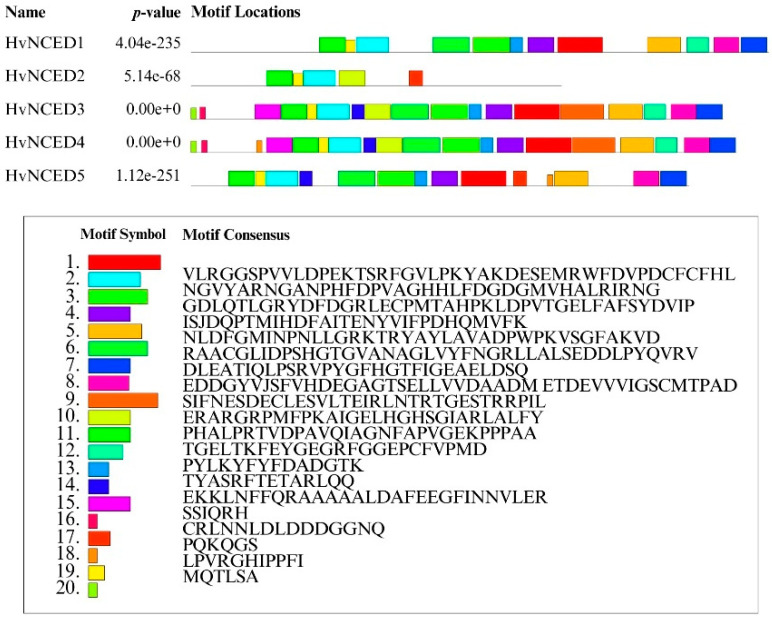
HvNCED protein’s conserved motifs. Different preserved motifs are represented by various colored boxes.

Out of the five *HvNCED* genes, only *HvNCED2* and *HvNCED5* have introns. *HvNCED2* has four introns, whereas *HvNCED5* has thirteen introns (Figure 5). The motif distribution study indicated that HvNCED1, HvNCED3, HvNCED4, and HvNCED5 possess a comparable number of motifs. HvNCED2 appears distinct from the others (Figure 6).

### 2.4. Expression Profile Analysis of the HvNCED Gene Family

**Figure 7:**
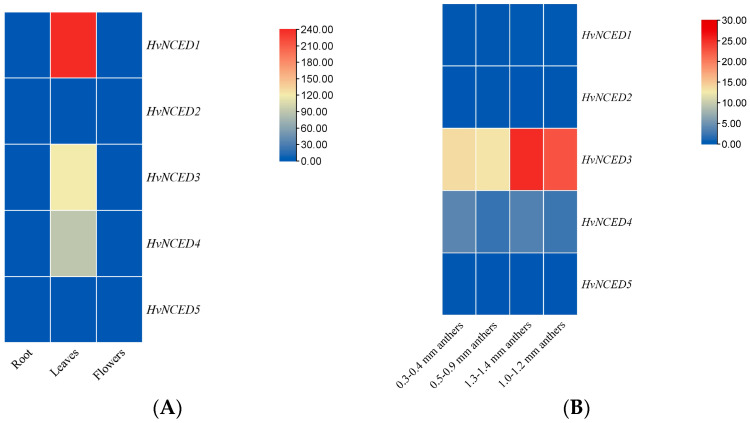
(**A**): Expression profile of *HvNCED*s in different tissues of *Hordeum vulgare* (root, leaves, and flowers). (**B**): Expression profile of *HvNCED*s in anthers at four specific time intervals (0.3–0.4 mm, 0.5–0.9 mm, 1.0–1.2 mm, 1.3–1.4 mm).

**Figure 8:**
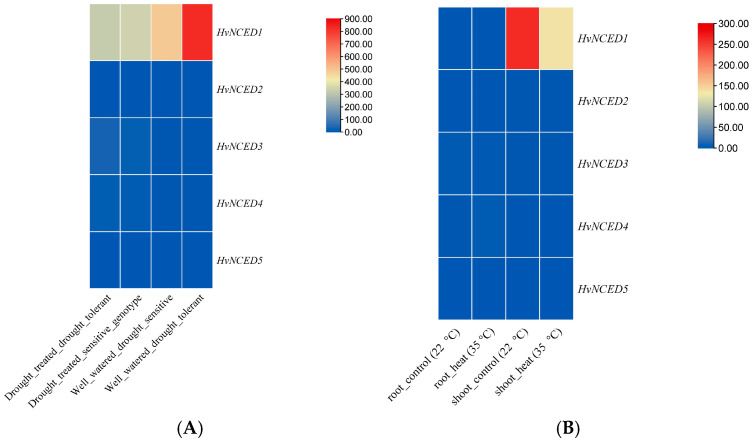
(**A**): Expression profile of *HvNCED*s across drought-tolerant and drought-sensitive barley genotypes. (**B**): Expression profile of *HvNCED*s across control (22 °C) and heat-stressed barley plants (35 °C).

The expression profile of *HvNCED*s in the root, leaves, and flowers of *H. vulgare* indicated a notable increase in expression for *HvNCED1* exclusively in the leaves. Both *HvNCED3* and *HvNCED4* exhibited an increase in expression levels in the leaves as well. The expression of the other *HvNCED*s (*HvNCED2* and *HvNCED5*) in the roots and flowers was nearly undetectable (Figure 7A). When examining the expression pattern of *HvNCED*s in anthers at four specific time intervals (0.3–0.4 mm, 0.5–0.9 mm, 1.0–1.2 mm, 1.3–1.4 mm), it was observed that *HvNCED3* exhibited a significant and noticeable upregulation in expression as the anthers developed (Figure 7B). The findings indicate that *HvNCED1*, *HvNCED3*, and *HvNCED4* are crucial in barley’s response to drought, with *HvNCED1* exhibiting the highest expression under well-watered conditions, while *HvNCED3* and *HvNCED4* show increased activity during periods of drought stress. In contrast, *HvNCED2* and *HvNCED5* show no expression under any conditions, indicating their lack of contribution to drought (Figure 8A). The expression levels of *HvNCED* genes in barley were analyzed under different temperature conditions, specifically comparing root and shoot responses at control (22 °C) and heat stress (35 °C) temperatures. The data are presented in Figure 8B.

### 2.5. RT-qPCR Analysis of the HvNCED Gene Family in the Leaves of Hordeum vulgare Under Salt Stress

**Figure 9:**
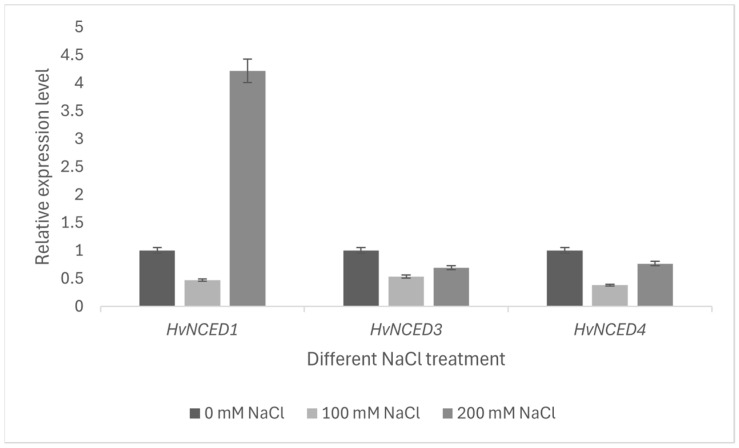
RT-qPCR analysis of the *HvNCED*s (*HvNCED1*, *HvNCED3*, *HvNCED4*) in the leaves of Hordeum *vulgare* under salt stress (*p* < 0.01).

The expression patterns of *HvNCED* genes in leaves subjected to salt stress were investigated (100–200 mM NaCl). No expression for *HvNCED2* and *HvNCED5* was detected, consistent with the expression profile of *HvNCED*s in the roots, leaves, and flowers of *H. vulgare* Only *HvNCED1* showed an elevation in expression levels compared to the expression under treatment with enhanced salt stress (200 mM NaCl) (*p* < 0.01), while the other two showed a decrease (Figure 9).

## 3. Discussion

Barley is a prominent cereal crop that holds the fourth position in global output, following maize, wheat, and rice [50]. Salinity, a common abiotic stressor, exerts a substantial impact on both plant growth and crop productivity [51]. Consequently, it is crucial to investigate the reaction and tolerance mechanism of *H. vulgare* to salt stress in order to enhance its tolerance. ABA is a crucial signaling molecule in plants when they are experiencing stress. Plant ABA relies on the crucial role of NCED enzymes, and this is accomplished by reducing 9-cis xanthophylls into ABA precursor xanthoxin. A limited set of *NCED* genes regulates the production of ABA. The *NCED* family is a very small gene family found in various species, including *A. thaliana, S. lycopersicum*, cotton (*Gossypium hirsutum*), and other crops [32,52,53].

This study identified five *HvNCED*s, consistent with *A. thaliana* and other grass family crops, indicating a stable evolutionary connection [42]. This suggests a rather stable evolutionary connection between members of this gene family. The *NCED* gene family in barley exhibits structural variability, as indicated by the differences in length, isoelectric points, and molecular weights of the HvNCED proteins. The phylogenetic analysis revealed three distinct clades among *NCED* genes from different species (Arabidopsis (*A. thaliana)*, rice (*O. sativa*), and *H. vulgare*), providing evidence for a conserved evolutionary relationship among these genes, suggesting that they have preserved their functional roles across species without notable expansion.

Gene duplication, particularly between *HvNCED3* and *HvNCED4*, highlights the significance of duplication in the *NCED* family expansion, with purifying selection indicated by Ka/Ks ratios below 1. The lack of data for *HvNCED5* and *HvNCED1* suggests insufficient synonymous substitutions for meaningful evolutionary metrics. This indicates that these genes are likely maintaining essential functions important for barley’s response to environmental stresses. The relatively higher Ks values for *HvNCED4* compared to *HvNCED2*, as well as for *HvNCED3* in relation to *HvNCED2*, suggest that these gene pairs have undergone more recent divergence than the other pairs analyzed. The synteny analysis identified conserved gene pairs across *H. vulgare, A. thaliana*, and *O. sativa*, suggesting a potential for functional conservation for these identified genes. The chromosomal localization of *HvNCED* genes offers insights into genomic structure and putative regulatory mechanisms. The examination of the exon-intron architectures of *HvNCED* genes revealed that some structural characteristics were conserved, although differences may suggest specific functions, as shown in *HvNCED*3 and *HvNCED*4.

In *A. thaliana,* all five AtNCEDs are uniquely localized to plastids, but their association with the thylakoid membrane varies [32]. In *Phaseolus vulgaris*, the PvNCED1 protein undergoes transport into chloroplasts and becomes associated with thylakoids [53]. In addition, the N-terminal region of Cowpea1’s (*Vigna unguiculata*) VuNCED1 protein directs sGFP into protoplast plastids, indicating that it acts as a transit peptide. Subcellular localization predictions offer valuable insights into the potential roles of these proteins in various cellular compartments. The presence of HvNCED1 within the plastid suggests a role in ABA biosynthesis, whereas the membrane localization of HvNCED2 and HvNCED5 implies possible functions in signal transduction pathways. The cytoplasmic localization of HvNCED3 and HvNCED4 suggests their involvement in cellular metabolism or stress response functions. The examination of cis-elements associated with hormones in the promoter region of *HvNCED* genes highlights their role in diverse hormonal responses. *HvNCED1*, *HvNCED2*, and *HvNCED3* play a significant role in the response to ABA, while also exhibiting regulatory features related to auxin and methyl jasmonate responsiveness. *HvNCED4* demonstrates an extensive array of cis-acting elements, demonstrating responsiveness not only to ABA, auxin, and MeJA, but also to gibberellin. In a comparable manner, *HvNCED5* is associated with responsiveness to ABA and MeJA. The findings underscore the complex regulatory networks of the *HvNCED* genes as they respond to various plant hormones and stress signals.

No expression of *HvNCED2* and *HvNCED5* was observed, consistent with the expression profile of *HvNCED*s in the roots, leaves, and flowers of *H. vulgare.* The expression levels of *HvNCED2* and *HvNCED5* in the roots and flowers were nearly undetectable. This may be attributed to gene expressions particular to developmental stages or environmental stress responses other than salinity. The presence of introns in these genes, which distinguishes them from other *NCED*s, along with their unique motif distribution and phylogenetic placement, indicates that they may have specialized functions. Additionally, their predicted membrane localization further supports the idea of distinct roles for *HvNCED*2 and *HvNCED5*. The substantial increase detected in leaves suggests the role of *HvNCED1* in leaf-specific functions or stress responses. In addition, the marginal elevation in the expression of *HvNCED3* and *HvNCED4* observed in leaves indicates their potential involvement in leaf-specific functions or stress responses.

Prior studies on tomato have shown *SlNCED*1’s role in ABA-mediated processes in pollen/anther metabolism and gene expression [54]. ABA also regulates pollen maturation by influencing anther-specific gene expression [55]. We analyzed *HvNCED* expression in anthers at four intervals (0.3–1.4 mm), finding significant upregulation of *HvNCED3* during development, suggesting its role in ABA-mediated anther/pollen development. The absence of *HvNCED3* expression in flowers, despite its elevated expression in anthers, can be attributed to temporal expression patterns. Gene expression can be modulated over time. *HvNCED3* may be expressed at a certain developmental phase of the anthers, but not during the flowering stage when floral gene expression was assessed. *HvNCED1’*s increased expression under well-watered conditions highlights its role in physiological functions and stress hormone modulation. Some genes remain active in barley roots and leaves under adequate water, indicating drought preparedness [56]. In contrast, *HvNCED3* and *HvNCED4* increase during drought, essential for adaptive mechanisms, while *HvNCED2* and *HvNCED5* show no expression, indicating limited drought tolerance contribution. Variations in gene expression among genotypes suggest a genetic basis for drought tolerance, with drought-tolerant varieties showing consistent expression profiles.

The study of *HvNCED* genes under varying temperatures reveals distinct expression patterns, with *HvNCED1* showing notable expression in shoots under normal conditions but reduced expression under heat stress. *HvNCED2* showed no expression, while *HvNCED4’*s increased expression under heat stress suggests its role in temperature adaptation. Previous studies indicate *NCEDs* regulate plant responses to environmental stressors, with overexpression of certain *NCED* genes improving drought tolerance [57,58,59,60,61]. However, not all *NCED*s respond similarly to stress; for example, in *A. thaliana NCED3* primarily responds to drought, while *NCED4* reacts to heat stress in lettuce [32,62]. The wheat *NCED1* gene is induced by various stressors and enhances water retention in transgenic tobacco [63].

In our study, we examined three *HvNCED* genes (*HvNCED1*, *HvNCED3*, *HvNCED4*) under salt stress (100–200 mM NaCl). Only *HvNCED1* showed upregulation at 200 mM NaCl, suggesting its significant role in ABA-dependent gene regulation related to salt stress. This aligns with findings that upregulating ABA regulatory genes enhances stress tolerance. For instance, transgenic plants expressing *VuNCED1* survived better under stress [64]. *OsNCED5* also increased under salt and water stress, regulating ABA-dependent gene expression [65]. A complexity in plant responses to salt was identified, highlighting a distinction between osmotic and ionic stress responses in saline conditions [66]. The prompt reaction to the external osmotic potential in saline circumstances is osmotic stress, whereas a delayed reaction occurs in ionic stress, resulting from the buildup of detrimental ions within the plant [66]. Therefore, the salt-specific gene list was linked to “response to auxin”, whereas the osmotic gene list was associated with “response to water deprivation” and “response to ABA” [66]. The downregulation of *HvNCED1* at 100 mM may reflect osmotic stress, while its upregulation at 200 mM indicates ionic stress adaptation. Conversely, the downregulation of *HvNCED3* and *HvNCED4* under salt stress suggests regulatory mechanisms affecting ABA levels. Their similar expression patterns imply a functional relationship, likely due to shared cytoplasmic locations and evolutionary history.

## 4. Materials and Methods

### 4.1. Plant Materials

Seeds of barley (local cultivar) were collected from the Seed and Seedling Center, Al-Baha, Saudi Arabia. Barley seedlings were sown in soil and maintained at a temperature of 22 °C during the day and night. The humidity level was consistently maintained at 60%. There were a total of 24 pots, each containing one plant, organized in a completely randomized manner with 3 blocks. Each block consisted of 8 pots, and every 2 pots inside a block were designated a biological replication. Following a three-month period, all plants were watered with distilled water containing varying quantities of NaCl (0 mM as a control, 100 mM, or 200 mM) every six days. A saucer was placed underneath the containers to preserve the moisture of the soil. After around 20 days of treatment, the deleterious impacts of salt on plants were apparent. Thus, each plant’s leaves were harvested separately. During this period, various symptoms indicative of salinity stress were monitored, including leaf chlorosis, wilting, premature leaf senescence, and reduced overall growth. The leaves chosen for expression analysis were those positioned at the 3rd leaf from the top on the selected plant, as this location signifies a younger, actively growing leaf.

#### Identification and Sequencing Analysis of the *HvNCED* Family Genes in *H. vulgare*

Annotations and genomic sequences of *H. vulgare* were obtained from the National Centre for Biotechnology Information (https://www.ncbi.nlm.nih.gov/datasets/genome/GCF_904849725.1/ (accessed on 1 September 2024)). The information about the *NCED* gene family of *A. thaliana* was retrieved from The Arabidopsis Information Resource (TAIR) database (https://www.arabidopsis.org/ (accessed on 1 September 2024)) and used as a query in the HMMER (3.3.2) software for creating the hidden Markov model profile in order to identify the *NCED* gene family in *H. vulgare* The HMMER software was employed to identify the NCED proteins of *H. vulgare* using this model. To verify that the candidates were NCED members, the SMART and Pfam databases were used. This eliminated redundant sequences and retained just those with the (PF03055) domain (https://www.ebi.ac.uk/interpro/search/text/ (accessed on 1 September 2024)) (UniProt, 2021).

### 4.2. Phylogenetic Analysis, Gene Duplication, and Chromosomal Location

Protein sequences for *NCED* genes from Arabidopsis (*A. thaliana*), rice (*O. sativa*), and *H. vulgare* were used to create the phylogenetic tree. The alignment of all NCED sequences was conducted using MAFFT v7.402 [67]. The phylogenetic analyses were performed using the raxmlGUI software 2.0 with 1000 bootstrap repeats and the GTR + G model for maximum likelihood estimate [68]. The TBtools software 1.6 was utilized to determine the values of Ka, Ks, and Ka/Ks [69]. The divergence time (T) was calculated using the formula T = Ks/(2 × 6.1 × 10^−9^) 10^−6^ million years ago (Mya). Paralogous genes with a Ka/Ks ratio greater than 1 were strongly positive, those around 1 were considered neutral, and those below 1 were indicative of purifying selection. The TBtools tool was used to build a chromosomal distribution and collinear *HvNCED* gene map based on data from the HvNCED family genes. The synteny study of the *NCED* gene in *H. vulgare* L., *A. thaliana*, and *O. sativa* was performed using the TBtools software. Afterwards, the results were displayed using a dual Synteny Plot.

### 4.3. Sequence Analysis and Features of Proteins and Genes

All HvNCED-identified protein sequences were validated by a conserved domain search (accession number: PF03055) using the Conserved Domain Database (https://www.ncbi.nlm.nih.gov/cdd (accessed on 1 September 2024)) [70]. To display *HvNCED* gene structures, the Gene Structure Display Server 2.0 (GSDS; http://gsds.cbi.pku.edu.cn (accessed on 1 September 2024)) was utilized. The hypothesized subcellular locations of HvNCED proteins were identified by means of the Plant-mSubP web application (https://bioinfo.usu.edu/Plant-mSubP (accessed on 1 September 2024)) [71]. Using the default settings, MEME server v 5.0.5 (http://meme-suite.org/tools/meme (accessed on 1 September 2024)) was used to analyze conserved motifs [72].

### 4.4. Cis-Regulatory Element Analysis of HvNCED Genes Promoters

To analyze the promoter regions, a 1.5-kbp upstream promoter sequence of the *HvNCED* genes was utilized. The Plant CARE database (http://bioinformatics.psb.ugent.be/webtools/plantcare/html/ (accessed on 1 September 2024)) was utilized for the estimation of cis-regulatory elements (CREs). Only hormone-related responsive elements were retained. TBtools was utilized to show the graphical locations of CREs within sequences [73].

### 4.5. Expression Profile Analysis of the HvNCED Gene Family

In order to analyze the expression patterns of *HvNCED*s in different tissues, data from the barley expression database (BarleyExpDB) were extracted [74]. This data were obtained from different projects, namely PRJEB50400, PRJNA744021, PRJNA752285, PRJEB40905, PRJNA324116, and PRJNA558196. Additional information regarding the details of the experiments is available at http://barleyexp.com/common.html (accessed on 1 September 2024). The log2-transformed FPKM values were utilized to create heatmaps illustrating the expression of *HvNCED*s. The heatmaps were generated using TBtools.

### 4.6. RNA Isolation and Gene Expression Analysis of the HvNCED Gene Familyin the Leaves Under Salt Stress

RNA was isolated from leaves using Trizol reagent following the manufacturer’s instructions. Gel electrophoresis was employed to evaluate the integrity of RNA. The RNA levels in the samples were measured using the NanoDrop 2000 Spectrophotometer (Thermo Scientific, UK). The Roche Light Cycler 96 System was used to perform RT-qPCR. The expression levels of *HvNCED1*, *HvNCED3*, and *HvNCED4* were quantified in 10 ng RNA samples using the Luna Universal One-Step RT-qPCR Kit from NEB.

To conduct RT-qPCR analysis, forward and reverse primers for the genes were designed (Table S1). The ^2−∆∆^CT method was employed to calculate the relative expression of *HvNCED*s in comparison to the expression levels of the housekeeping gene ACTIN (NCBI accession no: XP_044949308.1). The cycle threshold (Ct) values were derived from three independent biological replicates. To determine the statistical significance of the variation in gene expression between control and stress conditions, a two-tailed unpaired t-test with a *p* < 0.01 was performed.

### 4.7. Statistical Analysis

Statistical analyses were performed to evaluate the significance of alterations in gene expression levels under various salt stress conditions. The data were analyzed using a two-tailed unpaired t-test to compare the expression levels of *HvNCED* genes between the control group (0 mM NaCl) and the treated groups (100 mM and 200 mM NaCl). A *p*-value less than 0.01 (*p* < 0.01) was considered statistically significant. The results were presented as mean values with standard error bars to illustrate the variation among biological replicates. This methodology allowed us to assess the impact of salt stress on the expression of *HvNCED* genes and to confirm the validity of our findings.

## 5. Conclusions

Through a comprehensive examination of the *H. vulgare* genome, a total of five *HvNCED* genes have been identified. These genes were categorized into three distinct clades based on the results of phylogenetic analysis. The examination of motif and gene structure provided additional evidence supporting the accuracy of the classification. Evolutionary analysis revealed the homology of *NCED* genes between *A. thaliana* and *O. sativa,* indicating a conserved evolutionary relationship and potential functional conservation across different plant species.

Based on this study, HvNCED1 is located in the plastid and is mostly involved in ABA biosynthesis, whereas HvNCED2 and HvNCED5 are related to membrane functions, and HvNCED3 and HvNCED4 are located in the cytoplasm. The expression patterns of *HvNCED* genes under salt stress demonstrate that *HvNCED1* is upregulated at elevated salinity levels, highlighting its crucial function in modulating ABA-dependent responses to salt stress. In contrast, downregulation is shown by *HvNCED3* and *HvNCED4*, suggesting that a complex regulatory mechanism is involved in response to varying stress conditions. The lack of expression of *HvNCED2* and *HvNCED5* in *H. vulgare* suggests that these genes may be specifically regulated by developmental stages or environmental stressors other than salinity. Further research is necessary to understand the roles and regulation of these two genes in different contexts.

The results highlight the possibility of utilizing *HvNCED1* to improve salt tolerance in barley, given that elevated expression of *NCED* genes has been associated with enhanced stress resistance in various other species. The initial downregulation of *HvNCED1* is proposed as a response to osmotic stress, while its later upregulation is suggested to correspond to ionic stress, reflecting adaptive mechanisms in barley.

In conclusion, valuable insights into the role of *NCED* genes in barley’s response to salt stress are provided by this study, with an emphasis placed on the potential for genetic modifications to enhance crop resilience against abiotic stressors. Enhanced agricultural practices and crop management strategies could be achieved through further exploration of the regulatory mechanisms governing these genes. Future research should measure total carotenoids and ABA levels in samples to understand *H. vulgare*’s physiological responses under salt stress. This will help understand regulatory pathways and develop strategies for enhancing salt tolerance in crops, ultimately improving agricultural practices in saline conditions.

## Figures and Tables

**Table 1:** The identification of the members of the *HvNCED* gene family in *Hordeum vulgar* and their sequence analysis.

Accession id (NCBI)	Gene Name	Chromosome	Gene Length	CDS (bp)	Protein Length (aa)	Protein Molecular Weight (kDa)	Ip	GRAVY	No of Introns	Subcellular Localization
XP_044951576.1	*HvNCED1*	6H	2998	1932	643	69.17	6.24	−0.2	-	Plastid
XP_044961465.1	*HvNCED2*	7H	2308	1242	413	44.58	7.32	−0.04	4	Membrane
XP_044984628.1	*HvNCED3*	5H	2412	1797	592	64.60	5.4	−0.19	-	Cytoplasm
XP_044946847.1	*HvNCED4*	5H	2546	1824	607	65.98	5.34	−0.26	-	Cytoplasm
XP_044983988.1	*HvNCED5*	4H	5353	1668	555	62.73	5.8	−0.35	13	Membrane

**Table 2:** Ka/Ks ratios and estimated divergence time for expected paralogous *HvNCED* genes (NaN: not a number).

Seq_1	Seq_2	Ka	Ks	Ka_Ks
*HvNCED3*	*HvNCED4*	0.14107	0.426416	0.330826
*HvNCED1*	*HvNCED5*	0.696456	NaN	NaN
*HvNCED2*	*HvNCED4*	0.648955	1.271244	0.510488
*HvNCED2*	*HvNCED3*	0.553017	1.096642	0.504282

## Data Availability

All the data in this study are available upon request by contacting the corresponding author.

## Supplementary Materials

The following supporting information can be downloaded at: https://www.mdpi.com/article/10.3390/plants13233327/s1, Table S1: Primers used for RT-qPCR analysis of genes.
