# Supplementary material for: A Comprehensive Analysis of the 9-Cis Epoxy Carotenoid Dioxygenase Gene Family and Their Responses to Salt Stress in Hordeum vulgare L
Source: Plants (Basel). 2024 Nov 27;13(23):3327. doi: 10.3390/plants13233327 (PMC11644444; doi:10.3390/plants13233327)
Supplement: Supplementary file 1 [file plants-13-03327-s001.zip › plants-3260499-supplementary.pdf]

Table S1: Primers used for RT-qPCR analysis of genes

Primer' name	Sequence
HvNCED1F	GTCTTGCCCAACGTCTTCT
HvNCED1Rev	CCTCCATCGGGTTCATCTG
HvNCED2F	GGATCATGCTTGGCACAATATC
HvNCED2Rev	GCCTCCATCATCGTTCATCT
HvNCED3F	CTTCGCCATCACCGAGAATTA
HvNCED3Rev	GTCTTCTCCTTGTCGAGCAC
HvNCED4F	TGCTTCTGTTTCCACCTCTG
HvNCED4Rev	CGTCCGTGTCGTTGAAGAT
HvNCED5F	GCCATAGACTTTCCTCGAATCA
HvNCED5Rev	AAGATGCCACTCACCTTCAC
HvACTINfor	CGTGTTGGATTCTGGTGATG
HvACTINrev	AGCCACATATGCGAGCTTCT
